# May–Thurner syndrome, a diagnosis to consider in young males with no risk factors: a case report and review of the literature

**DOI:** 10.1186/s13256-021-02730-8

**Published:** 2021-03-19

**Authors:** Joel Zhen Khang Hng, Shu Su, Noel Atkinson

**Affiliations:** 1 Department of Vascular Surgery, The Royal Melbourne Hospital, 300 Grattan Street, Parkville, VIC 3050 Australia; 2 Department of Radiology, The Royal Melbourne Hospital, 300 Grattan Street, Parkville, VIC 3050 Australia

**Keywords:** May–Thurner syndrome, Left common iliac vein, Case report, Deep vein thrombosis, Risk factors, Ultrasound, Computer tomography venography, Endovascular, Stent, Venous hypertension

## Abstract

**Background:**

May–Thurner syndrome is an anatomical condition characterized by compression of the left common iliac vein by the right common iliac artery, causing venous outflow obstruction. It is an uncommon cause of deep vein thrombosis and is more prevalent among women. This paper highlights the importance of considering May–Thurner syndrome in young males without risk factors presenting with left lower limb pain, as endovascular treatment may be required.

**Case presentation:**

A 23 year-old Caucasian male presented with a 1-week history of left lower limb pain, edema, and pallor. He was found to have an unprovoked deep vein thrombosis on Doppler ultrasound involving the left fibular, soleus, gastrocnemius, popliteal, femoral, common femoral, and external iliac veins. A heparin infusion was commenced as the initial treatment for deep vein thrombosis. Further investigation with computer tomography pulmonary angiogram and computer tomography venography of the abdomen and pelvis showed bilateral pulmonary emboli and left common iliac vein compression with left common, internal, and external iliac vein thrombosis. He was diagnosed with May–Thurner syndrome despite having no risk factors. A retrievable Cook Celect Platinum inferior vena cava filter was placed, and thrombus of the left common femoral, external, and common iliac veins was treated successfully with AngioJet thrombectomy, thrombolysis using 200,000 units of urokinase, angioplasty and stenting using two Cook Zilver Vena venous self-expanding stents. Therapeutic enoxaparin was commenced on discharge. His filter was removed after 10 weeks. Hematological follow-up 4 months later showed an overall negative thrombophilia screen, and anticoagulation was switched to apixaban. He has had no recurrent thrombosis.

**Conclusions:**

Clinicians should have a low threshold to investigate for May-Thurner syndrome in patients with left lower limb venous thrombotic events regardless of risk factors, as endovascular treatment may be required to minimize the long-term sequelae of deep vein thrombosis. Duplex ultrasound can be used initially for diagnosis, and computer tomography venography used subsequently if the common iliac vein is not visualized on ultrasound. Endovascular treatment is preferred over anticoagulation alone, especially in otherwise fit patients presenting early, the aim being to reduce the chances of chronic venous hypertension in the lower limb.

## Background

May–Thurner syndrome (MTS) is a vascular condition caused by compression of the left common iliac vein by the right common iliac artery against the fifth lumbar vertebra, which may cause deep vein thrombosis (DVT) in the left lower limb [1]. Individuals with MTS are typically asymptomatic and may not be diagnosed in their lifetime, and the majority are female [2, 4]. MTS is an uncommon cause of DVT, accounting for approximately 2–5% of lower-extremity venous disorders [3, 4]. Risk factors for MTS include female sex, especially those postpartum, multiparity, use of oral contraceptives, scoliosis, dehydration, and hypercoagulable disorders [2–6]. There are very few reports in the literature of MTS in young males. Bhadra *et al.* described a rare case of a 21-year-old man who presented with bilateral pulmonary emboli and left lower limb DVT, who was found to have MTS and was treated with anticoagulation only [7]. Noninvasive treatment such as anticoagulation is usually sufficient to treat most DVTs. However, endovascular therapy to treat DVT is recommended in individuals with MTS to prevent recurrent thrombosis [3, 4, 8, 9]. This case report shows the importance of considering MTS as a diagnosis in young males without any risk factors presenting with lower limb pain, as more invasive treatment may be required to reduce the long-term sequelae of DVT, such as chronic venous hypertension and recurrent thrombosis.

## Case presentation

**Fig. 1:**
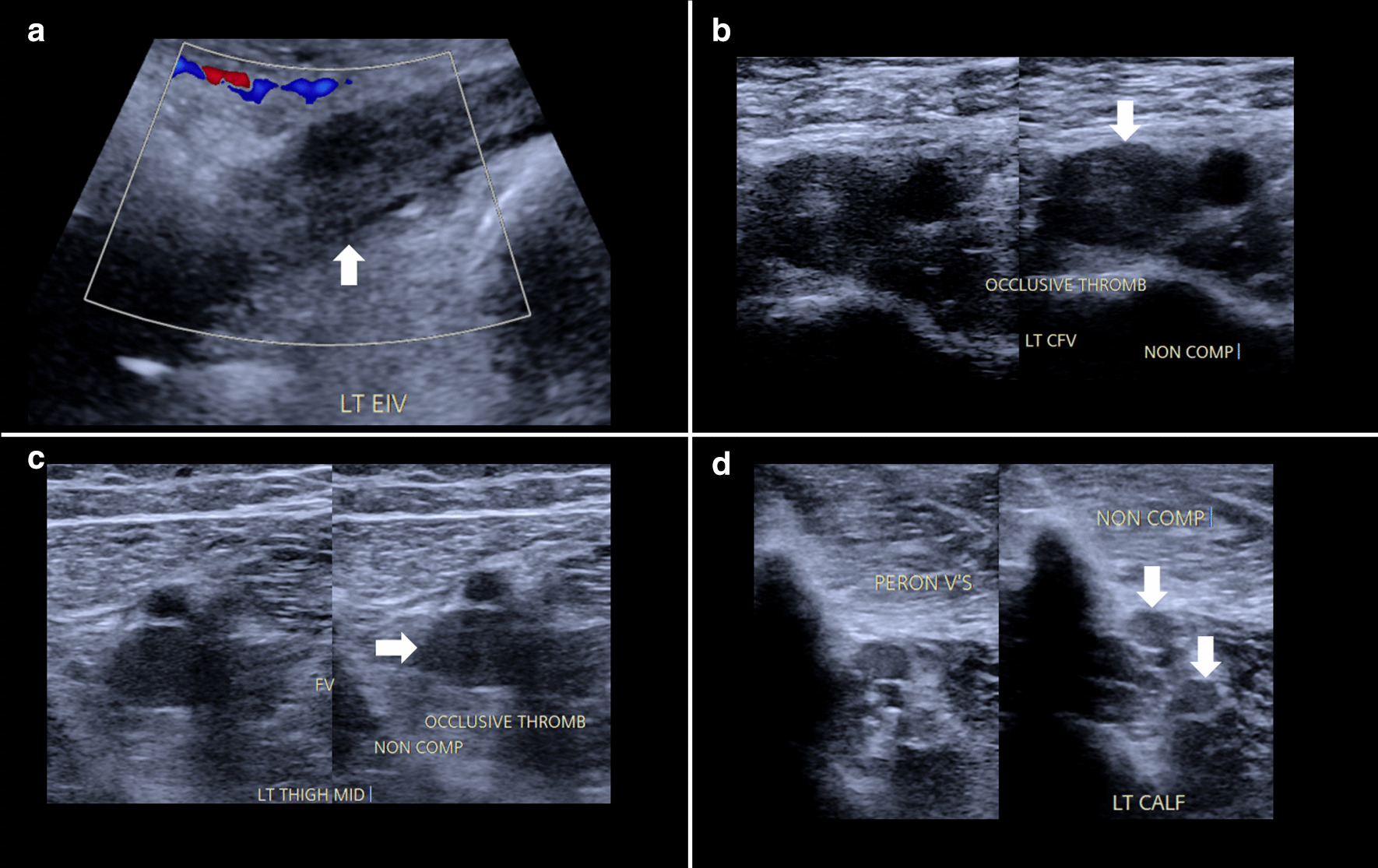
Absent Doppler flow within an occlusive thrombus-filled (arrow) left external iliac vein (**a**). Non-compressive occlusive thrombus (arrow) within the left common femoral, femoral, and fibular veins (**b**–**d** respectively)

A 23-year-old Caucasian male presented to a rural hospital with a 1-week history of left lower limb pain, edema, and pallor. His symptoms commenced 1 week prior to hospital admission, starting with pain in the entire left lower limb, and then edema, with the thigh worse than the lower leg, and finally pallor of the entire left lower limb. He denied any trauma, history of thromboembolic disease, shortness of breath, chest pain, alcohol consumption, smoking history, recreational drug use, or family history of hypercoagulable disorders. Past medical history included type 2 diabetes mellitus, which was managed with oral gliclazide MR 120 mg once daily and oral metformin XR 1000 mg once daily. He has no other significant medical history or family history and was not on any other regular medications. He was unemployed and lived in a rural town with a friend. On clinical examination, his left thigh was more swollen than his lower leg, with associated tenderness and pallor. The left femoral, popliteal, tibial and dorsalis pedis pulses were palpable. His lower-limb neurological examination was normal, with intact sensation and 5/5 power in all movements bilaterally. He had a heart rate of 85 beats per minute, blood pressure of 120/70 mmHg, oxygen saturation of 98% on room air, respiratory rate of 16 breaths per minute, and was afebrile at 36.5 ℃. Initial assessment with computed tomography (CT) angiography demonstrated no significant arterial disease. He was then found to have extensive DVT over the entire length of the left lower limb on venous Doppler ultrasound (US), involving the left fibular, soleus, and gastrocnemius veins up to the popliteal vein, femoral vein, common femoral vein, and external iliac vein (Fig. 1). He had normal renal function, serum urea, electrolyte levels, creatine kinase, calcium, magnesium, phosphate, and full blood examination. He had elevated serum C-reactive protein at 59 mg/L (normal range <10), a slightly elevated fibrinogen at 5.0 g/L (normal range 1.5–4.0 g/L), and mildly high gamma-glutamyl transferase of 107 U/L (normal range <65 U/L) and alkaline phosphatase of 135 U/L (normal range 30–110 U/L). Otherwise, his coagulation profile and liver function test were normal. Plasma D-dimer was 12.62 µg/ml fibrinogen-equivalent units (normal range <0.50). The initial impression of the clinical picture was phlegmasia alba dolens. He was given an intravenous heparin loading dose of 5000 units and then commenced on an intravenous heparin infusion at a rate of 1080 units per hour, titrated according to the activated partial thromboplastin time (target range of 60–85 seconds) and transferred to the vascular surgery unit of a metropolitan hospital for further management.

**Fig. 2:**
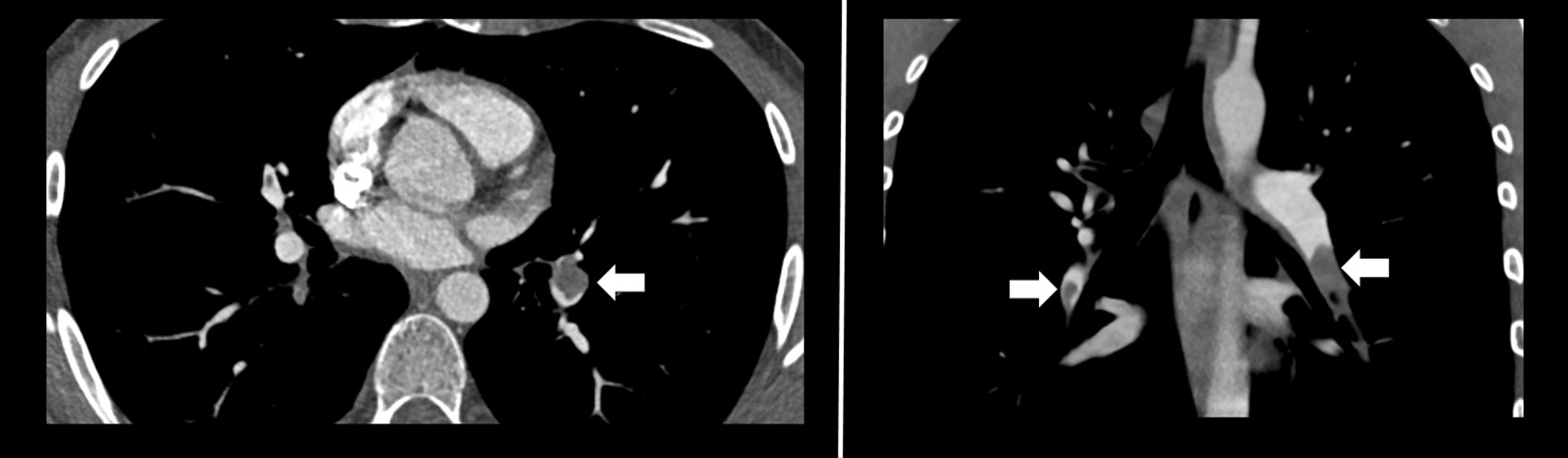
Bilateral pulmonary arterial emboli (arrows)

**Fig. 3:**
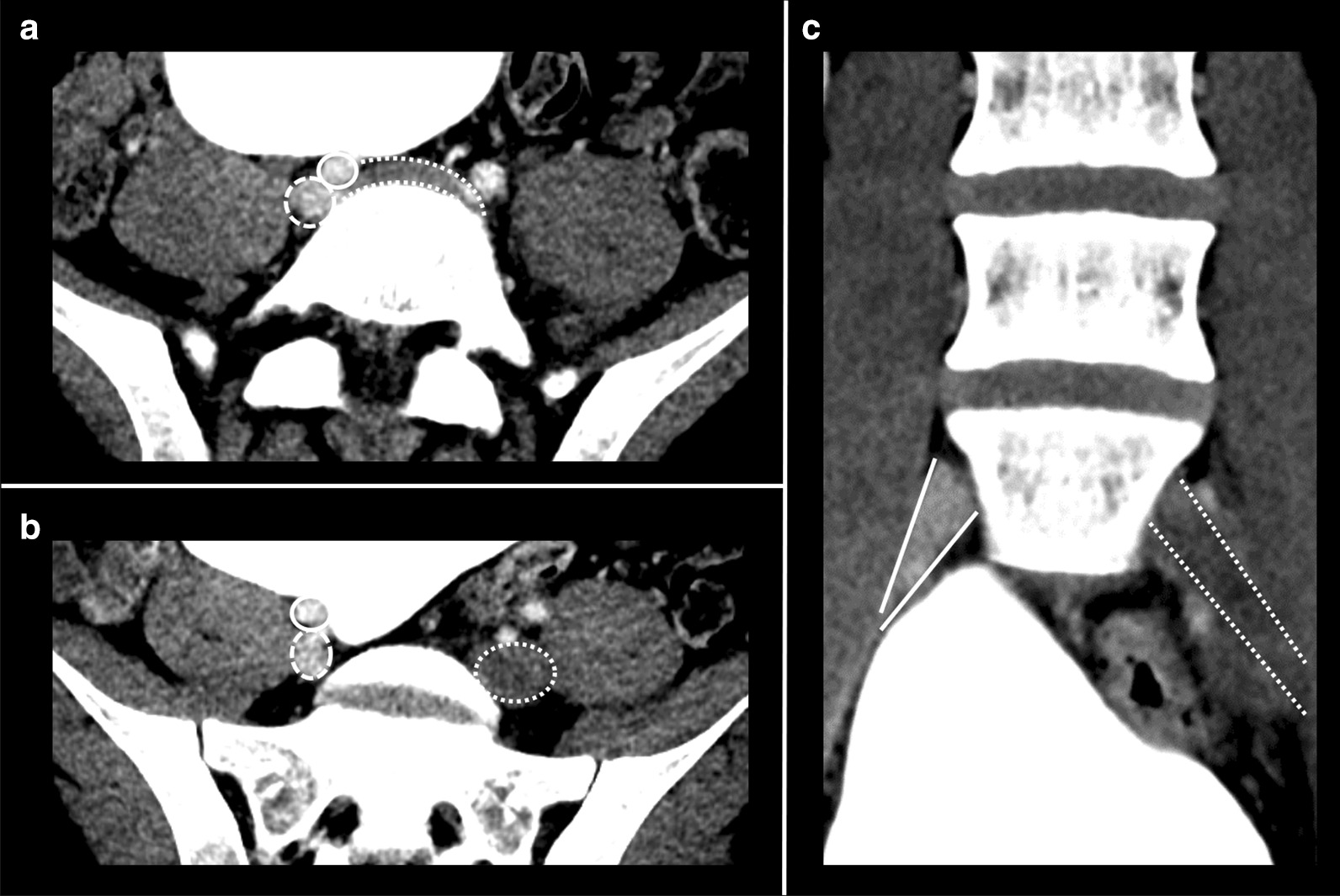
CT venogram demonstrating right common iliac artery (solid outline) compressing the left common iliac vein (dotted outline), in keeping with May–Thurner syndrome (**a**). The left common iliac vein (dotted outline) is distended with thrombus and does not enhance compared with the right common iliac vein (dashed outline) (**b**, **c**)

**Fig. 4:**
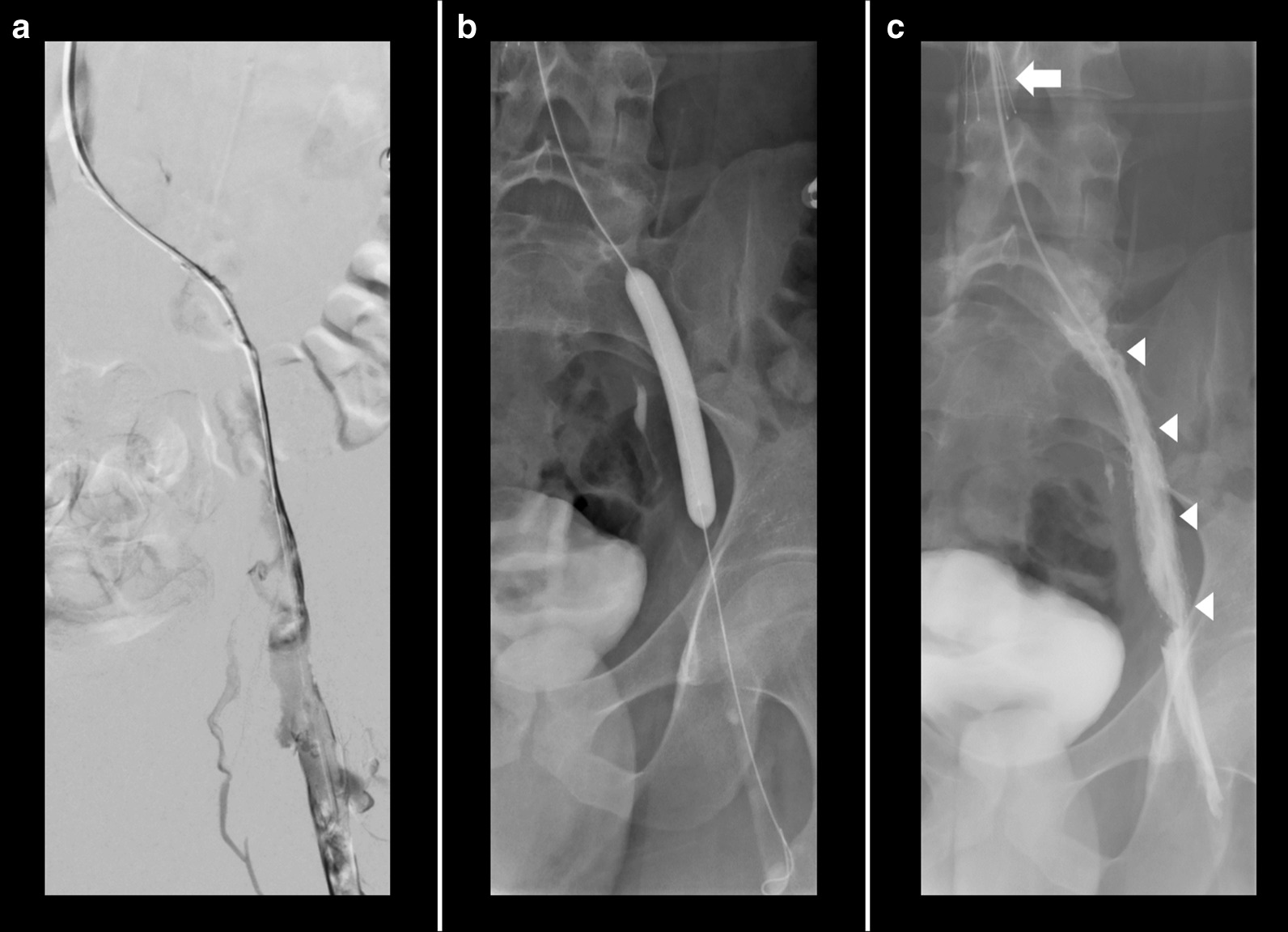
Left common iliac vein thrombus demonstrated on catheter venogram (**a**), recanalized following angioplasty (**b**). Cook Celect Platinum IVC filter (solid arrow) and left common iliac vein Cook Zilver Vena stents (arrow heads) were inserted (**c**).

Further investigation with CT pulmonary angiogram and CT venography of the abdomen and pelvis showed bilateral interlobar and segmental pulmonary emboli, and compression of the left common iliac vein with an extensive thrombus of the left common iliac, internal, and external iliac veins, consistent with MTS (Figs. 2 and 3). The Interventional Radiology team subsequently inserted a temporary Cook Celect Platinum inferior vena cava (IVC) filter via the right internal jugular vein. The left common iliac vein was cannulated, and venography showed an extensive thrombus across the left common femoral, external, and common iliac veins. AngioJet mechanical thrombectomy, thrombolysis using 200,000 units of urokinase, angioplasty, and stenting of the left common femoral, external, and common iliac veins, using two Cook Zilver Vena venous self-expanding stents of 16-mm diameter and 63-mm length each were all performed successfully (Fig. 4). On discharge from hospital, he wore left lower limb compression stockings, and the heparin infusion was switched to subcutaneous therapeutic enoxaparin 60 units twice per day. Oral anticoagulation was not used because of its slightly higher risk of future thrombosis, when compared with enoxaparin. He received intravenous heparin infusion for 4 days. His IVC filter was removed 10 weeks later without complication.

Therapeutic enoxaparin was continued until a follow-up appointment at the hematology clinic 4 months later. His thrombophilia screen was overall negative, with a negative prothrombin gene mutation, normal protein C, protein S, lupus anticoagulant, anti-beta-2 glycoprotein 1, and anticardiolipin antibody. He had marginally low antithrombin at 73% (normal range 80–120%); however, this may have been related to the presence of thrombosis or heparin therapy at the time of testing, rather than an underlying genetic disorder. Factor V Leiden will be tested at his next appointment. He was advised to remain on anticoagulation until next follow-up and was switched from therapeutic enoxaparin to oral apixaban 5 mg twice per day because of convenience of use despite a slightly higher risk of future clots. He has had no recurrent thrombosis of his lower limb and has a follow-up due in 4 months.

## Discussion

Our case report describes an unusual presentation of MTS in a young male with no risk factors. The majority of the current literature are about MTS in females with risk factors, and only a few discuss it in males. The approach to diagnosis and treatment has often been debated and the literature vary. This case report is unique because it shows an unexpected and unusual presentation of MTS that has not been documented much in literature and establishes a proper approach to diagnosis and treatment. It suggests that MTS may be underdiagnosed and highlights the importance of identifying these patients, as more invasive treatment is required to reduce the risk of chronic venous hypertension.

The prevalence of MTS is estimated to be more than 20% in the general population; Kibbe *et al.* have even suggested that iliac vein compression could be a normal anatomic pattern [1, 2]. However, MTS only accounts for 2–5% of lower limb venous disorders [3, 5]. Some publications have found that up to 50% of iliofemoral DVT cases can have iliac vein stenosis caused by extrinsic venous compression and propose that MTS may be a common condition that is underdiagnosed [8, 9]. Other studies propose this may be because MTS is overshadowed by more common risk factors for DVT such as hypercoagulability, female sex, and history of oral contraceptive use [3, 10]. Interestingly, risk factors for MTS include female sex, especially those who are postpartum, multiparity, patients with a history of oral contraceptive use, scoliosis, dehydration, and hypercoagulable disorders [2–6]. The overlap of risk factors between DVTs and MTS could be a reason why MTS is overlooked, as many clinicians may attribute DVTs to these risk factors instead of an underlying anatomical pathology. Our case report is rare, in that the patient is a young male who did not have any of the risk factors mentioned above.

Bhadra *et al.* described a case of a 21-year-old-male who presented with bilateral PE and left lower limb DVT, who was found to have MTS despite having no risk factors and was treated with anticoagulation only [7]. They advocated clinicians to have a low threshold for suspecting MTS in young patients with unprovoked left lower limb DVT, and to consider endovascular treatment in MTS. Our case report also highlights the importance of considering MTS in young males presenting with left lower limb pain, and additionally discusses the approach to diagnose MTS as well as strongly advocates for endovascular treatment in symptomatic MTS to minimize the long-term sequelae of DVT.

The approach to investigating for MTS varies amongst clinicians. Some publications recommend investigating patients with risk factors for MTS [6, 11]. However, as our case report has noted, MTS can occur in young males with no risk factors. Several studies suggest that the approach to diagnosis of MTS should be a combination of clinical presentation despite risk factors, and imaging findings; that is, if a patient presents with lower limb swelling or pain, clinicians should investigate for DVT and also consider MTS as a cause [3–5, 8, 9, 12]. Our case report justifies these suggestions.

Duplex US should be performed as a first-line investigation as it is noninvasive and can assess dynamically for both DVT and MTS. However, visualization of the common iliac vein using duplex US was estimated to be only 47%, limited by variables such as sonographer experience, patient body habitus, and the deep location of iliac veins in the pelvis [3, 5, 6, 9, 12]. This was the case with our patient, whereby the most proximal vein adequately studied by venous Doppler US was the left external iliac vein.

CT venography can be considered if duplex US does not adequately assess the left common iliac vein. CT venography, however, exposes patients to ionizing radiation, and timing of venous phase contrast can be difficult, which can lead to suboptimal images [5, 8]. However, in several recent articles, CT venography has been considered sufficient to detect left common iliac vein compression [9, 12, 13]. The benefits of CT venography also include the detection of other causes of venous compression, such as a pelvic mass, and Chung *et al.* also demonstrated that anatomical abnormalities in the iliofemoral region can be evaluated by CT venography [12–14]. Note that many studies on CT venography in MTS contain relatively small sample sizes, and further studies with larger sample sizes are required to determine the efficacy of CT venography in detecting MTS. In our case report, MTS was adequately diagnosed on CT, which further justifies the use of CT in diagnosing MTS.

As MTS is an anatomical issue, endovascular treatment to correct the mechanical obstruction together with anticoagulation therapy is preferred over anticoagulation alone in patients with symptomatic MTS [4, 5, 8–10, 12, 13, 15]. Endovascular treatment consists of thrombectomy, thrombolysis, angioplasty, and eventually stent placement, as was the case with our patient. Patients with symptomatic MTS that do not have the mechanical obstruction treated, have a high thrombosis recurrence rate, up to 73% according to a publication by the Radiological Society of North America in 2014 [13]. Treating the mechanical obstruction aims to prevent the long-term sequelae of iliofemoral DVT such as chronic venous hypertension, which can lead to recurrent thrombosis. A study evaluating the long-term outcomes of patients with iliofemoral thrombosis managed without endovascular treatment concluded that more than 40% of the patients developed venous hypertension and overall impaired venous outflow 5 years later [16]. Incomplete clearance of an iliofemoral thrombus also leads to a significantly higher risk of recurrent thrombosis and postthrombotic syndrome [17]. Conversely, a study assessing the effects of AngioJet thrombectomy in DVT demonstrated that more than 90% of patients with complete thrombus clearance remained patent after 12 months [18]. These publications highlight the importance of endovascular treatment to ensure complete clot clearance and to prevent chronic venous hypertension for better long-term outcomes.

It is often debated whether stent placement is necessary, as the stent itself may become stenosed. However, a recent prospective study by Gabr and Allam evaluated stent patency in 61 symptomatic patients with MTS, and showed an overall primary patency rate of 93% at 1 year after treatment [15]. Patients who have undergone angioplasty alone have been shown to have a lower patency rate than those who were stented [8, 9, 12]. The use of anticoagulation post endovascular treatment for at least 6–12 months to prevent rethrombosis is generally agreed upon [4, 5, 8, 13]. Overall, current literature suggests that endovascular treatment such as stenting is safe, at least for the short term. Our patient has had no stenosis of the stent, rethrombosis, or symptoms since treatment, which supports stent placement and continuing anticoagulation in patients with symptomatic MTS.

In our case report, the anticoagulation of choice chosen post initial DVT treatment was therapeutic enoxaparin because of the opinion that it is more effective at preventing thrombosis than oral anticoagulants such as apixaban. However, a recent literature review showed that most direct oral anticoagulants, including apixaban had an efficacy and safety profile similar to that of enoxaparin in treating patients with venous thromboembolism [19]. Hence, the choice of anticoagulation regime post initial DVT treatment should depend on other factors, such as practicality of use, individual risk, clinician experience, and availability.

IVC filters should not be kept for a long period of time because of risk of filter-related DVT [20]. Prolonged implantation of filters is also associated with high retrieval failure rates. A prospective study on retrievable IVC filters found a high retrieval failure rate of 40.9% after 7 months of implantation [20]. Based on current literature, it is recommended that IVC filters be removed as soon as possible once clinically safe from pulmonary embolism to prevent complications with retrieval. This time frame varies among clinicians but should be within 25–54 days of implantation [21]. Our patient did not suffer from any IVC filter-related complications.

## Conclusion

Patients presenting with left lower limb venous thrombotic events should be investigated for MTS using duplex US and/or CT venography, especially if no other causes for symptoms are found. This is particularly important as endovascular treatment, including stenting of the obstructed common iliac vein, is preferred over anticoagulation alone in managing May–Thurner syndrome, to prevent chronic lower limb venous hypertension and thus recurrent thrombosis.

## Data Availability

All data generated or analyzed during this study are included in this published article.

## Abbreviations

MTS: May–Thurner syndrome
DVT: Deep vein thrombosis
CT: Computer tomography
US: Ultrasound
IVC: Inferior vena cava
