# Correction to: May–Thurner syndrome, a diagnosis to consider in young males with no risk factors: a case report and review of the literature

**DOI:** 10.1186/s13256-021-03195-5

**Published:** 2021-11-26

**Authors:** Joel Hng, Shu Su, Noel Atkinson

**Affiliations:** 1 Department of Vascular Surgery, The Royal Melbourne Hospital, 300 Grattan Street, Parkville, VIC 3050 Australia; 2 Department of Radiology, The Royal Melbourne Hospital, 300 Grattan Street, Parkville, VIC 3050 Australia

## Correction to: J Med Case Reports (2021) 15:141 https://doi.org/10.1186/s13256-021-02730-8

Following publication of the original article [1], the authors identified an error in the author name Joel Hng.

The incorrect author name is: Joel Zhen Khang Hng

The correct author name is: Joel Hng

The author group has been updated above and the original article [1] has been corrected.
